# CircHADHA-augmented autophagy suppresses tumor growth of colon cancer by regulating autophagy-related gene *via* miR-361

**DOI:** 10.3389/fonc.2022.937209

**Published:** 2022-10-13

**Authors:** Ying Shi, Jinying Li, Ming Tang, Jingwen Liu, Yalu Zhong, Wei Huang

**Affiliations:** ^1^ Department of Gastroenterology, The First Affiliated Hospital, Jinan University, Guangzhou, China; ^2^ Department of Gastroenterology, Hepatology and Infectious Diseases, Internal Medicine I, Tübingen University Hospital, Tübingen, Germany; ^3^ Endoscopy Center, The First Affiliated Hospital, Jinan University, Guangzhou, China

**Keywords:** circHADHA, colon cancer, asymptomatic polyp, tumor growth, autophagy

## Abstract

Colon cancer undergoes a traditional pathway from colon polyps to colon cancer. It is of great significance to investigate the key molecules involved in carcinogenesis from polyps to malignancies. Circular RNAs (circRNAs) are stably expressed in human body fluids such as plasma. Here, we demonstrated a differential expression pattern of plasma circRNAs in healthy individuals, colon polyp patients and colon cancer patients using circRNA Arraystar microarray. We explored that circRNA HADHA (circHADHA) was upregulated in plasma from polyp patients, whereas it was downregulated in plasma from colon cancer patients. Overexpression of circHADHA promoted autophagy in colon epithelial cells. Moreover, in colon cancer cells, overexpression of circHADHA promoted autophagy, whereas it inhibited cell proliferation and colony formation. CircHADHA increased the expression of ATG13 *via* miR-361 in both colon epithelial and cancer cells. ATG13 knockdown reduced autophagy even in the presence of circHADHA in colon cancer cells. Furthermore, the growth of circHADHA-overexpressing colon cancer cell-derived *xenograft* tumors was significantly decreased compared with control tumors in nude mice. In conclusion, circHADHA was differentially expressed in the plasma of healthy individuals, colon polyp patients and colon cancer patients. CircHADHA promoted autophagy by regulating ATG13 *via* miR-361 in both colon epithelial and cancer cells. CircHADHA suppressed tumor growth by inducing cell autophagy in colon cancer cells. CircHADHA potentially serves as a biomarker for screening of precursor colon cancer and a therapeutic target for colon cancer treatment.

## Introduction

Colon cancer is the third leading cause of cancer-related new cases and death (1, 2). In China, colon cancer is also one of the most common cancers (3). Colon cancer undergoes a series of processes from normal colon epithelial cells to aberrant crypt foci and finally to malignancy. Colon polyps are precursor lesions of colon cancer in the conventional adenoma-to-carcinoma pathway, in which oncogenic transformation is driven by mutations in *APC, KRAS, SMAD4*, and *TP53* (4–6). Except colonoscopy, conventional screening methods for screening and early diagnosis of colon cancer include blood tests, fecal occult blood testing (FOBT), fecal immunochemical test (FIT), and DNA or RNA stool tests (7). However, novel noninvasive and economical technologies and biomarkers remain to be explored to combine colonoscopy diagnosis for the prediction of the malignant transformation from premalignancy to colon cancer (8–10).

Circular RNAs (circRNAs) are a class of endogenous RNAs with a special circular covalently bonded structure (11). Unlike linear RNA, circRNAs exhibit resistance to digestion by ribonucleases, such as RNase R, due to lack of 3’ and 5’ terminals (12, 13). CircRNAs also have a longer half-life (12). With the development of RNA sequencing technologies and bioinformatics, the dynamic expression patterns and diversity of circRNAs were identified in a variety of diseases, including cancer (14, 15). In recent years, circRNA-based liquid biopsy biomarkers have gained much attention (16). CircRNAs are highly abundant in blood and enriched in plasma exosomes, serving as potential biomarkers for the prediction and diagnosis of colon cancer (17–19). In addition, more biological functions of circRNA were revealed, such as acting as microRNA (miRNA) sponges (20), modulating the expression of parental genes (21), regulating alternative splicing (22), being protein scaffolds, and being involved in RNA–protein interactions (23).

Here, we investigated the dynamic expression pattern of plasma circRNA in the malignant transformation from colon polyps to colon cancers and revealed the biological behavior of circHADHA in colon epithelial and cancer cells. Our results indicated that circHADHA may serve as a biomarker for premalignancy prediction and potential therapeutics for colon cancer patients.

## Materials and methods

### Patients

All participants were adults (≥18 years of age). Healthy individuals were eligible if they were excluded from colon polyps by colonoscopy and had no other diseases by medical checkups. The colon polyp group included asymptomatic populations that were diagnosed with colorectal polyps by colonoscopy screening, excluding patients with colorectal cancer and other comorbidities by medical checkups. The histological diagnosis of the enrolled patients with colon polyps included hyperplastic polyps, tubular adenoma, villous adenoma, and tubulovillous adenoma. The colon cancer group included patients with primary colorectal cancer diagnosed for the first time by colonoscopy and histology who had not yet undergone surgical resection and drug treatment, excluding patients with other comorbidities.

### Plasma sample collection and circRNA hybridization

Blood samples were collected from colon cancer and polyp patients and healthy individuals from the First Affiliated Hospital, Jinan University. All samples from colon cancer and polyp patients were collected before medical treatment. Plasma was isolated from blood by centrifuging at RCF 1,500g for 10 min. Total RNA was extracted from each plasma sample and prepared according to the Arraystar’s standard protocols. The concentrations of the RNA samples were measured by NanoDrop ND-1000. The integrity of RNA was assessed by electrophoresis on a denaturing agarose gel. RNA from each sample was treated with Rnase R to degrade the abundant linear RNAs and enrich circRNAs. The enriched circRNAs were amplified and transcribed using a random priming (Arraystar Super RNA Labeling Kit; Arraystar). After complementary RNA (cRNA) was purified (RNeasy Mini Kit, Qiagen), the hybridization was performed on Human circRNA Array (Arraystar Inc.). Agilent Scanner G2505C was used for array scanning.

### Data analysis of circRNA array

Fold changes were computed between the groups for each circRNA. The statistical significance of the difference may be conveniently estimated by Student’s t-test. Fold changes >1.5 and *P* < 0.05 were statistical significance. R software/limma package (24) was used for differential expression of the microarray data.

### CeRNA network analysis

The potential interaction of messenger RNA (mRNA) and miRNA with circRNA was predicted (Arraystar’s home-made miRNA target prediction software) based on TargetScan and miRanda databases. The competing endogenous RNA (ceRNA) network was illustrated by Cytoscape 3.0.

### Cell culture

Human colon epithelial cells HCoEpiC purchased from ScienCell Research Laboratories (Carlsbad, CA, USA) were cultured in Dulbecco’s modified Eagle’s medium (Gibco) supplemented with 10% fetal bovine serum (Gibco) and 1% penicillin G/streptomycin (Gibco). NCM460 cells (25) were cultured in M3Base medium (INCELL) supplemented with 10% fetal bovine serum (Gibco). LoVo cells purchased from American Type Culture Collection (ATCC) (Manassas, VA, USA) were cultured in Ham’s F-12K (Kaighn’s) Medium (Gibco) supplemented with 10% fetal bovine serum (Gibco) and 1% penicillin G/streptomycin (Gibco). Cells were cultured at 37°C in an atmosphere of 95% air and 5% CO_2_.

### Generation of stable circHADHA-overexpressing cells

The human circHADHA-overexpressing construct was generated based on modified pLCDH-ciR vector (Geneseed Biotech). The head-to-tail splice junction in circHADHA was predicted and designed as ggtggaacccctgGCatgttagccgcttgcaaga. Stable circHADHA-overexpressing HCoEpiC, NCM460, and LoVo cells were selected by puromycin (Invitrogen). The expression level of circHADHA was measured using real-time PCR.

### Real-time PCR for circHADHA

Tissue cells were homogenized in TRIzol Reagent (Invitrogen). Chloroform was added to separate the homogenate into a clear upper aqueous layer, an interphase, and an organic layer. RNA was precipitated from the aqueous layer with isopropanol. First-strand cDNA was synthesized using Geneseed II First Strand cDNA Synthesis Kit (Geneseed Biotech). Real-time PCR was performed using Geneseed qPCR SYBR^®^ Green Master Mix (Geneseed Biotech). Divergent primers of circHADHA: forward primer, 5’-tggtggaacccctggcatgt-3’, and reverse primer, 5’-caggcaggatccattgatggc-3’.

### CircRNA fluorescence *in situ* hybridization

Cells were cultured on coverslips with 1.0 μg/ml of lipopolysaccharide (LPS) treatment. Cells were removed from the medium and rinsed with phosphate buffered saline (PBS). After incubating with 0.5% TritonX-100 at room temperature for 15 min, cells were fixed in 4% paraformaldehyde. The cells were washed in phosphate buffered saline (PBS), treated with 100% ethanol, and then air-dried. Briefly, digoxin-labeled probes against circHADHA (5’, 3’ fluorescein isothiocyanate (FITC)-labeled) and miR-361 (5’, 3’ Cy3-labeled) targets (Geneseed Biotech) were denatured at 85°C for 5 min and hybridized at 37°C overnight. On the following day, the slides were washed in 2× saline-sodium citrate buffer (SSC, Sigma-Aldrich). Subsequently, blocking was performed with 3% bovine serum albumin (BSA) at 37°C for 30 min, and the anti-digoxigenin fluorescence-conjugated antibodies were added to the slides at 37°C for 1 h. After washing in PBS, the cell nuclei were stained by 50 µl DAPI/Antifade solution (Sigma-Aldrich). Rubber cement (MP Biomedicals) was used for sealing coverslips, which were observed under the laser scanning confocal microscope afterward.

### LC3B autophagy assay

The sequence of LC3B-h was inserted into modified pmCherry vector. Cells were cultured on coverslips with LPS (1.0 μg/ml) treatment. Cells were removed from the medium and rinsed with PBS. After incubating with 0.5% TritonX-100 at room temperature for 15 min, cells were fixed in 4% paraformaldehyde. The cells were washed in PBS, treated with 100% ethanol, and then air-dried. Cell nuclei were stained by 50 µl DAPI/Antifade solution (Sigma-Aldrich). Rubber cement (MP Biomedicals) was used for sealing coverslips, which were observed under the laser scanning confocal microscope afterward.

### Dual-luciferase reporter assay in circHADHA and miRNA candidates

Briefly, circHADHA dual-luciferase reporter constructs were generated by inserting the total length of circHADHA or the mutations of the miRNA target sites in circHADHA fragment into psiCHECK-2 dual-luciferase vector (Promega). Two mutation fragments (MUT_1 and MUT_2) were designed for the target sites of hsa-miR26a-1, hsa-miR-26a-2, hsa-miR-361, and hsa-miR-214. All miRNA mimics, miRNA inhibitors, and corresponding controls were purchased from GenePharma. HCoEpiC were seeded in 24-well plates at a density of 6 × 10^4^/well. The dual-luciferase reporter constructs with wild-type or mutant circHADHA gene were cotransfected with miRNA mimic, inhibitor, or corresponding controls, respectively. Then, 48 h after cotransfection, the luminescence activity of both firefly and Renilla luciferase was analyzed using Dual-Luciferase Reporter Assay System (Promega).

### Dual-luciferase reporter assay in ATG13-3’UTR and miR-361

Briefly, ATG13-3’UTR dual-luciferase reporter constructs were generated by inserting the total length of ATG13-3’UTR segments or the mutations of the miR-361 target sites in the ATG13-3’UTR fragment into psiCHECK-2 dual-luciferase vector (Promega). HCoEpiC were seeded in 24-well plates at a density of 6 × 10^4^/well. The dual-luciferase reporter constructs with wild-type or mutant 3’UTR in ATG13 gene were cotransfected with miR-361 mimic, inhibitor, or corresponding controls (GenePharma), respectively. Then, 48 h after cotransfection, the luminescence activity of both firefly and Renilla luciferase was analyzed using Dual-Luciferase Reporter Assay System (Promega).

### Competitive inhibition assay

HCoEpiC with or without circHADHA overexpression were seeded in six-well plates at a density of 2 × 10^5^ cells/well. miR-361 mimic, inhibitor, or corresponding controls were transfected by HiPerFect Transfection Reagent (Qiagen). Then, 48 h after transfection, cells were homogenized in TRIzol Reagent (Invitrogen) and RNA was extracted by RNeasy Kits (Qiagen) according to the manufacturer’s instructions. First-strand cDNA was synthesized using the miScript II RT Kit (Qiagen). Real-time PCR was performed using QuantiTect SYBR Green PCR Kits (Qiagen). The primers target ATG13: forward primer: 5’-GGCAATTTGAGAGGACCCCA-3’; reverse primer: 5’-CAGTGTCCTCACCAGCAGTT-3’. The primers target GAPDH: forward primer: 5’-AGAAGGCTGGGGCTCATTTG-3’; reverse primer: 5’-GCAGGAGGCATTGCTGATGAT-3’.

### Western blot

Total proteins were extracted from cells and tissues using RIPA buffer (Thermo Scientific). The lysate was centrifuged, and the supernatant was immediately transferred to a fresh tube. The protein concentration was determined using the BCA Protein Assay kit (Thermo Scientific). The prepared cell lysate was added into 4× NuPAGE LDS sample buffer (Invitrogen) and boiled for 10 min. Samples were loaded into Mini-Protean TGX Precast Gels (4%–15%, Bio-Rad). The samples were run on a Mini-Protean TGX Precast Gel (4%–15%, Bio-Rad) and then transferred to polyvinylidene fluoride (PVDF) membranes in protein transfer buffer for 60 min. Following transfer, non-specific binding on the membrane was blocked, and the membrane was incubated with primary antibodies at 4°C overnight. After washing three times with TBST, the membrane was incubated with secondary antibodies at room temperature for 1 h. Antibodies against ATG13 (E1Y9V, 13468) and mTOR (7C10, 2983) were purchased from Cell Signaling Technology (Danvers, MA, USA). Antibodies against LC3B (ab48394), Beclin 1 (EPR19662, ab207612), p62 (EPR4844), and Bcl-2 (ab196495) were purchased from Abcam (Boston, MA, USA).

### The generation of shATG13

shRNA sequence (5’-GCCATGTTTGCTCCCAAGAAT-3’) for the Atg13 gene was designed by the algorithm of ThermoFisher (http://rnaidesigner.thermofisher.com/rnaiexpress/). shATG13 plasmid was generated and was transfected in LoVo cells by Lipofectamine 3000 (Thermo Fisher Scientific). The knockdown efficiency of shATG13 was measured by real-time PCR and Western blot analysis.

### Cell viability assay

Cells with stable circHADHA overexpression and corresponding control cells were seeded into 96-well plates at a density of 2 × 10^3^ cells/well in the presence or absence of LPS (1.0 μg/ml). After 24, 48, and 72 h, cells were incubated with 10 μl of Cell Counting Kit-8 solution (DoJinDo) for 4 h. The absorbance was measured using a microplate reader at a wavelength of 450 nm. Three independent experiments were performed in triplicate.

### Colony formation assay

Briefly, cells were seeded in six-well plates at a density of 500 cells/well and treated with or without LPS (1.0 μg/ml). The colonies were fixed in 4% paraformaldehyde and then stained in 1% crystal violet after a 14-day culture. The colonies containing over 50 cells were counted. Three independent experiments were performed in triplicate.

### Apoptosis assay

Apoptosis was evaluated using the Annexin V-FITC Apoptosis Detection Kit I (BD Biosciences), according to the manufacturer’s instructions. Cells were cultured in low-glucose DMEM (Gibco, Thermo Fisher Scientific) and treated with or without LPS (1.0 μg/ml) for 24 h. Then, cells were suspended in 500 μl of binding buffer and stained with 5 μl of Annexin V-fluorescein isothiocyanate and 2.5 μl of propidium iodide for 10 min at room temperature. The samples were subjected to flow cytometry. The data were analyzed using Summit software (FlowJo).

### TUNEL assay

TdT-mediated dUTP nick-end labeling (TUNEL) assays were performed using the one-step TUNEL kit (Beyotime Institute of Biotechnology) following the manufacturer’s instructions. Cells were cultured on poly-(L-lysine)-coated coverslips in 12-well plates in low-glucose DMEM (Gibco, Thermo Fisher Scientific) and treated with or without LPS (1.0 μg/ml) for 24 h. Cells were fixed in 4% paraformaldehyde and then permeabilized with 0.1% Triton X-100 before photophobic incubation in 50 μl TUNEL reaction mixture for 1 h at 37°C. Cell nuclei were stained with DAPI for 2 min at room temperature.

### Inflammatory cytokine assay

HCoEpiC and NCM460 cells with or without circHADHA overexpression were seeded in six-well plates at a density of 2 × 10^5^ cells/well with LPS (1.0 μg/ml) treatment. miR-361 was transfected by HiPerFect Transfection Reagent (Qiagen). Then, 24 h after transfection, cells were homogenized in TRIzol Reagent (Invitrogen), and RNA was extracted by RNeasy Kits (Qiagen) according to the manufacturer’s instructions. First-strand cDNA was synthesized using the miScript II RT Kit (Qiagen). Real-time PCR was performed using QuantiTect SYBR Green PCR Kits (Qiagen). The primers target IL1β: forward primer: 5’-AGGAAGATGCTGGTTCCCTG-3’; reverse primer: 5’-GCATCGTGCACATAAGCCTC-3’. The primers target IL17a: forward primer: 5’-CAAGAACTTCCCCCGGACTG-3’; reverse primer: 5’-CTCTCAGGGTCCTCATTGCG-3’. The primers target Toll-like receptor 4 (TLR4): forward primer: 5’-GCCATTGCTGCCAACATCAT-3’; reverse primer: 5’-ACTGCCAGGTCTGAGCAATC-3’. The primers target GAPDH: forward primer: 5’-AGAAGGCTGGGGCTCATTTG-3’; reverse primer: 5’-GCAGGAGGCATTGCTGATGAT-3’.

### The generation of subcutaneous xenografts in nude mice

LoVo control (Ctrl, 5 × 10^6^) and LoVo-circHADHA (circHADHA, 5 × 10^6^) cells were subcutaneously injected into nude mice. Three days later, solid tumors were observed in mice that received cell injections. The size of xenograft tumors was measured every 3 days using a Vernier caliper {[length (mm) × width (mm)^2^]/2}. The xenograft tumors were dissected and weighed after mice were sacrificed.

### Immunohistochemistry

Xenograft tumor tissues were fixed in formalin and embedded in paraffin before being sectioned. Antigen was retrieved by Citrate Antigen Retrieval solution (Maxim Biotech). Tissue sections were deparaffinized and rehydrated. The slides were treated with peroxidase and blocked with 10% serum for 2 h at room temperature. The slides were incubated with antibody against ATG13 (ab105392, Abcam) overnight at 4°C. On the following day, the sections were rinsed and then incubated with secondary antibodies (Maxim Biotech). DAB Detection Kit (Maxim Biotech) was applied to the slides before counterstaining with hematoxylin.

### Statistical analysis

Statistical analysis was performed using SPSS 21.0 software (SPSS Inc.). Data between two groups were compared by using Student’s t-test. Two-way ANOVA analysis was used for the comparison between multiple groups. The values are expressed as the mean ± standard deviation of at least three independent experiments performed in triplicate. *P* < 0.05 was considered to be statistically different. Graphs were plotted using GraphPad Prism 9.0 (GraphPad Software Inc.).

## Results

### CircRNA was differentially expressed in plasma from healthy individuals, colon polyp patients and colon cancer patients

**Figure 1:**
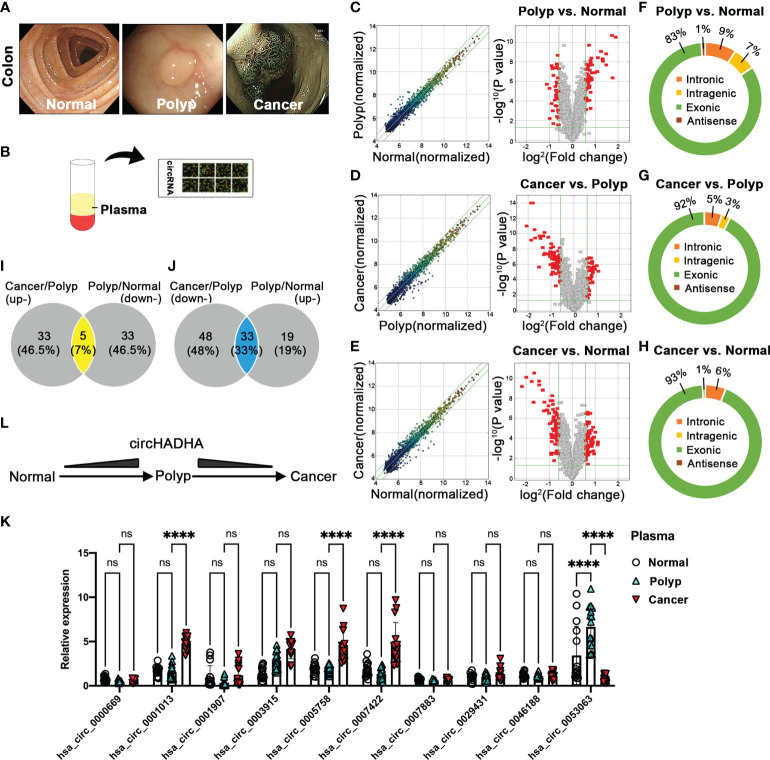
Plasma circRNA expression pattern in healthy individuals and colon polyp and colon cancer patients. **(A)** The endoscopic diagnosis of healthy individuals (left panel) and colon polyp (central panel) and colon cancer (right panel) patients. **(B)** The schematic diagram of Arraystar assay performance in plasma circRNAs. **(C)** The differential expression of plasma circRNAs in polyp patients (*N* = 5) and healthy individuals (*N* = 5). The expression variation of circRNAs was assessed by the scatter plot between polyp patients and healthy individuals (left panel). The green lines represented fold change lines. The circRNAs above the top green line and below the bottom green line indicated more than 1.5-fold change of circRNAs between the two compared samples. The differential expression of circRNAs was analyzed by volcano plots between polyp patients and healthy individuals (right panel). Red points represented the differentially expressed circRNAs with statistical significance (1.5-fold upregulation and downregulation, *P* < 0.05). **(D)** The differential expression of plasma circRNAs in colon cancer (*N* = 5) and colon polyp patients (*N* = 5). The expression variation of circRNAs was assessed by the scatter plot between colon cancer and colon polyp patients (left panel). The green lines represented fold change lines. The circRNAs above the top green line and below the bottom green line indicated more than 1.5-fold change of circRNAs between the two compared samples. The differential expression of circRNAs was analyzed by volcano plots between colon cancer and colon polyp patients (right panel). Red points represented the differentially expressed circRNAs with statistical significance (1.5-fold upregulation and downregulation, *P* < 0.05). **(E)** The differential expression of plasma circRNAs in colon cancer patients (*N* = 5) and healthy individuals (*N* = 5). The expression variation of circRNAs was assessed by the scatter plot between colon cancer patients and healthy individuals (left panel). The green lines represented fold change lines. The circRNAs above the top green line and below the bottom green line indicated more than 1.5-fold change of circRNAs between the two compared samples. The differential expression of circRNAs was analyzed by volcano plots between cancer patients and healthy individuals (right panel). Red points represented the differentially expressed circRNAs with statistical significance (1.5-fold upregulation and downregulation, *P* < 0.05). **(F–H)** The composition of types in detectable circRNAs. **(F)** The composition of circRNA types in polyp and colon cancer patients. **(I)** The Venn analysis between upregulated circRNAs from colon cancer patients compared with colon polyp patients and downregulated circRNAs from colon polyp patients compared with healthy individuals. **(J)** The Venn analysis between downregulated circRNAs from colon cancer patients compared with colon polyp patients and upregulated circRNAs from colon polyp patients compared with healthy individuals. **(K)** The potential circRNA candidates were validated by performing real-time PCR in plasma from healthy individuals (*N* = 15) and colon polyp (*N* = 15) and colon cancer (*N* = 15) patients. **(L)** The schematic diagram of circHADHA dynamic alteration in healthy individuals and colon polyp and colon cancer patients. ****P < 0.0001; ns, no significant difference.

We collected plasma from healthy individuals and colon polyp and colon cancer patients confirmed by endoscopic diagnosis (**Figure 1A**) and analyzed 2,162 human circRNAs by Arraystar (**Figure 1B**). Pairwise comparison indicated that 52 circRNAs were upregulated and 38 circRNAs were downregulated in the colon polyp group compared with healthy individuals (**Figure 1C**). In addition, 38 circRNAs were upregulated and 81 circRNAs were downregulated in the colon cancer group compared with colon polyps (**Figure 1D**). Among them, 29 circRNAs were upregulated while 37 circRNAs were downregulated in colon cancer groups compared with healthy individuals and colon polyps (**Figures 1D, E**). Most detectable candidates were predicated as exonic circRNAs (**Figures 1F–H**).

Then, we performed a further analysis between upregulated and downregulated circRNAs in plasma from different groups to explore potential indicators that evaluate the malignant transformation of colon cancer. Venn diagram demonstrated that five overlapping circRNAs were upregulated in the colon cancer group (colon cancer vs. colon polyp), while they were downregulated in the colon polyp group (colon polyp vs. healthy individuals) (**Figure 1I**; **Supplementary Table S1**). Thirty-three overlapping circRNAs were downregulated in colon cancer (colon cancer vs. colon polyp), whereas they were upregulated in the colon polyp group (colon polyp vs. healthy individuals) (**Figure 1J**; **Supplementary Table S1**). We validated 10 circRNA candidates with the most significance and performed real-time PCR to identify potential biomarkers (**Figure 1K**). Relative expression of candidate circRNAs demonstrated that hsa_circ_0053063 was upregulated in plasma from colon polyp patients compared with healthy individuals (*P* < 0.0001), whereas it was downregulated in plasma from colon cancer patients compared with colon polyp (*P* < 0.0001) (**Figures 1K, L** **)**. In addition, the expression of plasma hsa_circ_0001013 (*P* < 0.0001), hsa_circ_0005758 (*P* < 0.0001), and hsa_circ_0007422 (*P* < 0.0001) was increased in colon cancer compared with colon polyp (**Figure 1K**). Since gene symbol of circ_0053063 is HADHA (circBase database: http://www.circbase.org), we termed it as circHADHA. As a result, circHADHA may be a potential indicator for premalignant colon cancer.

### CircHADHA increased autophagy in colon epithelial cells

**Figure 2:**
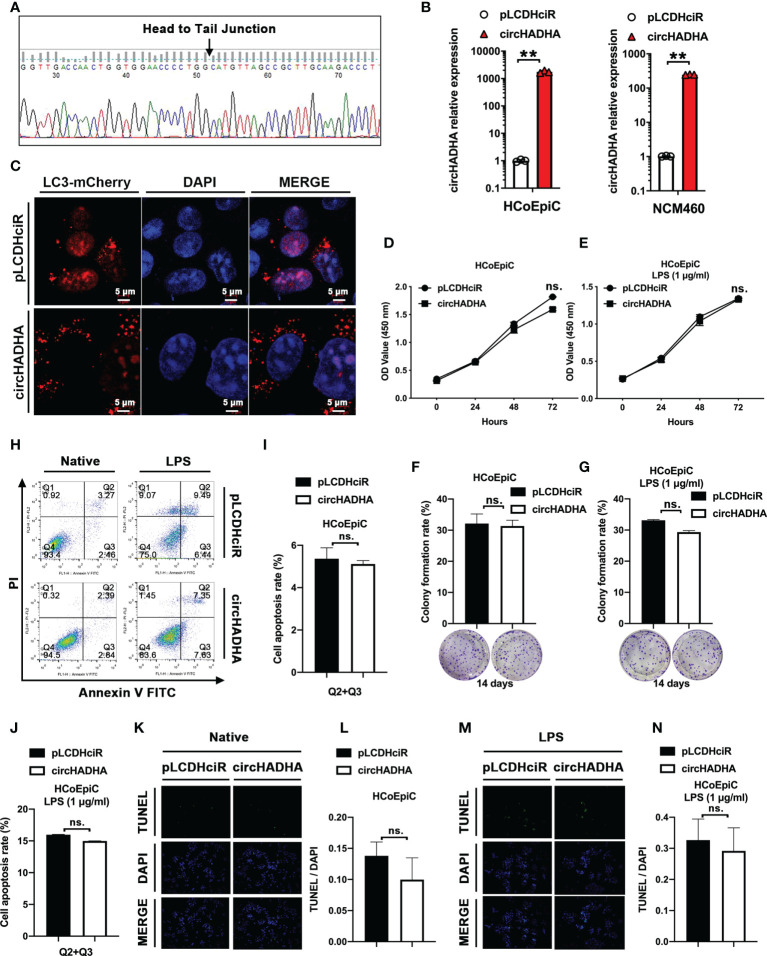
circHADHA increased autophagy in LPS-injured colon epithelial cells. **(A)** The sequence of head-to-tail splice junction in circHADHA. **(B)** The generation of circHADHA-overexpressing HCoEpiC (left panel) and NCM460 (right panel) cells. **(C)** The LC3B-mCherry-based autophagy assays in HCoEpiC. Overexpression of circHADHA promoted LC3B-positive autophagosomes compared with corresponding control in LPS-injured HCoEpiC. Cell autophagy was observed and imaged by the laser scanning confocal microscope. mCherry represented LC3B-positive autophagosomes. Cell nuclei were labeled with DAPI. **(D, E)** The performance of CCK-8 proliferation assays. Overexpression of circHADHA did not alter cell viabilities in HCoEpiC **(D)** and LPS-injured HCoEpiC **(E)**. **(F, G)** The performance of colony formation assays. The overexpression of circHADHA did not affect colony formation in HCoEpiC **(F)** and LPS-injured HCoEpiC **(G)** cells. **(H–J)** The performance of apoptosis by flow cytometry. Overexpression of circHADHA did not regulate apoptosis compared with corresponding control in HCoEpiC **(H, I)** and LPS-injured HCoEpiC **(H, J)**. **(K–N)** The performance of apoptosis by TUNEL assays. Overexpression of circHADHA did not change apoptosis compared with corresponding control in HCoEpiC **(K, L)** and LPS-injured HCoEpiC **(M, N)**. Three independent experiments were performed. **P < 0.01; ns, no significant difference.

In order to elucidate the roles of circHADHA in colon epithelial cells, we generated circHADHA-overexpressing HCoEpiC (*P* < 0.01) and NCM460 (*P* < 0.01) cells (**Figures 2A, B**). LPS was used to induce injury in colon epithelial cells with circHADHA overexpression or corresponding control. We expressed LC3B with mCherry in circHADHA-overexpressing and control cells to perform LPS-induced autophagy assays. We found that the overexpression of circHADHA significantly promoted mCherry-labeled LC3B-positive autophagosomes induced by LPS in HCoEpiC compared with controls (**Figure 2C**). We also performed CCK-8 and colony formation assays to explore the proliferation ability mediated by circHADHA in colon epithelial cells. We found that overexpression of circHADHA did not contribute to cell viability in HCoEpiC (**Figure 2D**) and LPS-injured HCoEpiC (**Figure 2E**). In addition, circHADHA overexpression did not affect colony formation in HCoEpiC (**Figure 2F**) and LPS-injured HCoEpiC (**Figure 2G**). We examined cell apoptosis by flow cytometry and found that circHADHA overexpression did not mediate the alteration of apoptosis in HCoEpiC (**Figures 2H, I** **)** and LPS-injured HCoEpiC (**Figures 2H, J** **)**. Moreover, TUNEL assays also demonstrated that circHADHA overexpression did not significantly regulate apoptosis compared with control in HCoEpiC (**Figures 2K, L** **)** and LPS-injured HCoEpiC (**Figures 2M, N** **)**.

### CircHADHA regulated autophagy and proliferation in colon cancer cells

**Figure 3:**
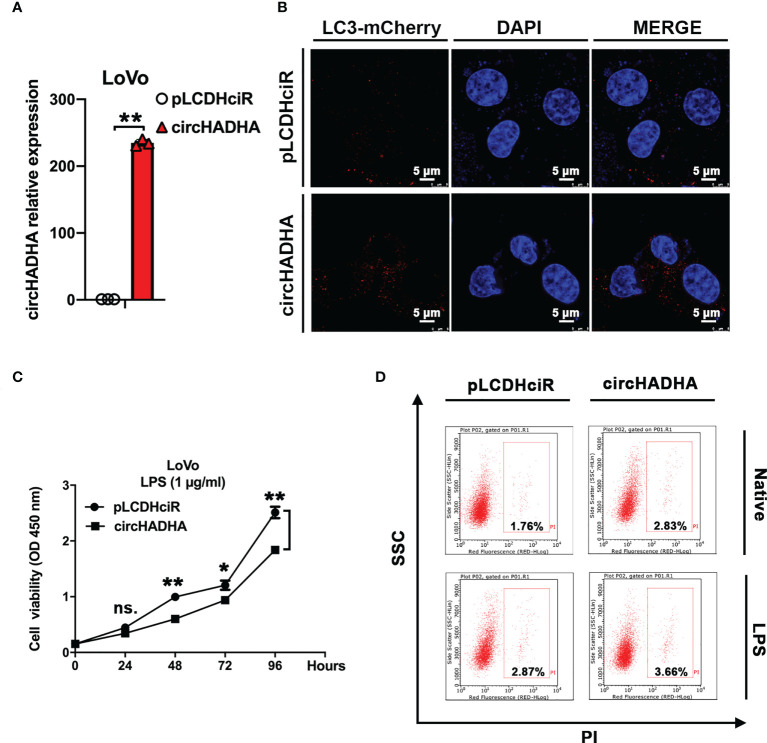
CircHADHA regulated autophagy and proliferation in colon cancer cells. **(A)** The generation of circHADHA overexpressed LoVo colon cancer cells. **(B)** The LC3B-mCherry-based autophagy assays in LoVo cells. Overexpression of circHADHA promoted autophagy compared with control in LPS-induced LoVo cells. mCherry represented LC3B-positive autophagosomes. Cell nuclei were labeled with DAPI. **(C)** The measurement of cell viability was performed by CCK-8 assay. Overexpression of circHADHA inhibited proliferation compared with control in LPS-induced LoVo colon cancer cells. **(D)** Cell apoptosis was performed by flow cytometry. No significant alteration of apoptosis was observed by overexpression of circHADHA compared with corresponding control in LoVo and LPS-induced LoVo cells. Three independent experiments were performed. **P < 0.01; *P < 0.05; ns, no significant difference.

Subsequently, we generated circHADHA-overexpressing LoVo colon cancer cells to investigate its behavior in colon cancer. The relative expression of circHADHA was significantly increased in circHADHA-overexpressing LoVo in comparison with control cells (**Figure 3A**, *P* < 0.01). We performed autophagy assays and found that overexpression of circHADHA increased mCherry-labeled LC3B-positive autophagosomes in LPS-induced LoVo compared with control cells (**Figure 3B**). We examined cell viability by CCK-8 assay and found that overexpression of circHADHA inhibited proliferation in LoVo and in LPS-induced LoVo compared with corresponding control cells after 48 h (*P* < 0.01), 72 h (*P* < 0.05), and 96 h (*P* < 0.01) (**Figure 3C**). Furthermore, we detected apoptosis and found that circHADHA overexpression did not significantly regulate apoptosis compared with control in LoVo and LPS-injured LoVo cells (**Figure 3D**).

### CircHADHA is a sponge that binds to miR-361 directly

**Figure 4:**
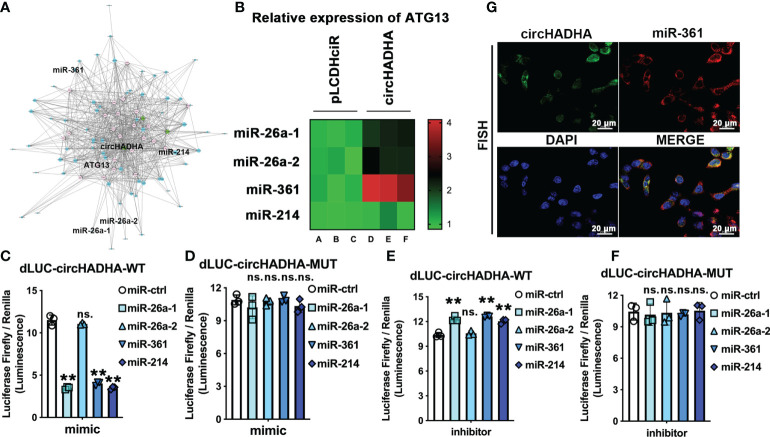
CircHADHA is a sponge that binds to miR-361 directly. **(A)** The potential binding candidates miR-26a-1, miR-26a-2, miR-361, miR-214, and ATG13 were predicted by ceRNA analysis for circHADHA to regulate autophagy. **(B)** The relative expression of ATG13 transfected by candidates miR-26a-1, miR-26a-2, miR-361, and miR-214 in HCoEpiC. miR-361 mediated the most significant increase of ATG13 expression with circHADHA overexpression compared to control in HCoEpiC. **(C–F)** The dual-luciferase reporter system with full-length and mutant circHADHA was generated. **(C)** Candidate miR-26a-1, miR-361, and miR-214 significantly decreased the luciferase reporter activities of full-length circHADHA with the transfection of miRNA mimics. **(D)** The miRNA candidates did not affect the luciferase reporter activities of mutant circHADHA with the transfection of miRNA mimics. **(E)** Candidate miR-26a-1, miR-361, and miR-214 significantly increased the luciferase reporter activities of full-length circHADHA with the transfection of miRNA inhibitors. **(F)** The miRNA candidates did not affect the luciferase reporter activities of mutant circHADHA with the transfection of miRNA inhibitors. **(G)** FISH assay demonstrated a colocalization of circHADHA and miR-361 in HCoEpiC. FITC represented circHADHA. Cy3 represented miR-361. Cell nuclei were labeled with DAPI. Three independent experiments were performed. **P < 0.01; ns, no significant difference.

We analyzed ceRNAs to predict the candidates of RNAs that potentially interacted with circHADHA. Interactome analyses indicated that miR-26a-1, miR-26a-2, miR-361, and miR-214 were most related to circHADHA, and ATG13 related to both circHADHA and autophagy (**Figure 4A**; **Supplementary Table S2**). We transfected mimics of candidate miRNAs in circHADHA-overexpressing HCoEpiC and control cells and performed real-time PCR to measure ATG13 expression. We found that miR-26a-1, miR-26a-1, and miR-361 mimics increased the expression level of ATG13 in circHADHA-overexpressing HCoEpiC compared to control cells (**Figure 4B**). Among them, miR-361 resulted in the most significant increase of ATG13 expression in the presence of circHADHA in HCoEpiC (**Figure 4B**). Furthermore, we generated dual-luciferase (dual-LUC) reporter system with full-length or mutants of circHADHA and cotransfected with miRNAs. By performing the dual-LUC reporter assays, we found that the luciferase reporter activities of full-length circHADHA were significantly decreased by transfection with mimics of miR-26a-1 (*P* < 0.01), miR-361 (*P* < 0.01), and miR-214 (*P* < 0.01) compared to the transfection of control mimic in colon epithelial cells (**Figure 4C**), whereas the luciferase reporter activities of mutant circHADHA did not change by transfection with mimics of miRNA candidates compared to the control mimic (**Figure 4D**). On the contrary, the luciferase reporter activities of full-length circHADHA were significantly increased by transfection with inhibitors of miR-26a-1 (*P* < 0.01), miR-361 (*P* < 0.01), and miR-214 (*P* < 0.01) compared to the transfection of control inhibitor in colon epithelial cells (**Figure 4E**). While the luciferase reporter activities of mutant circHADHA did not change by transfection with inhibitors of miRNA candidates compared to the control inhibitor (**Figure 4F**). Moreover, we performed FISH assay and demonstrated a colocalization of circHADHA and miR-361 in HCoEpiC (**Figure 4G**). As a result, circHADHA is a sponge that binds to miR-361 directly.

### CircHADHA released ATG13 inhibition by competitively recruiting miR-361

**Figure 5:**
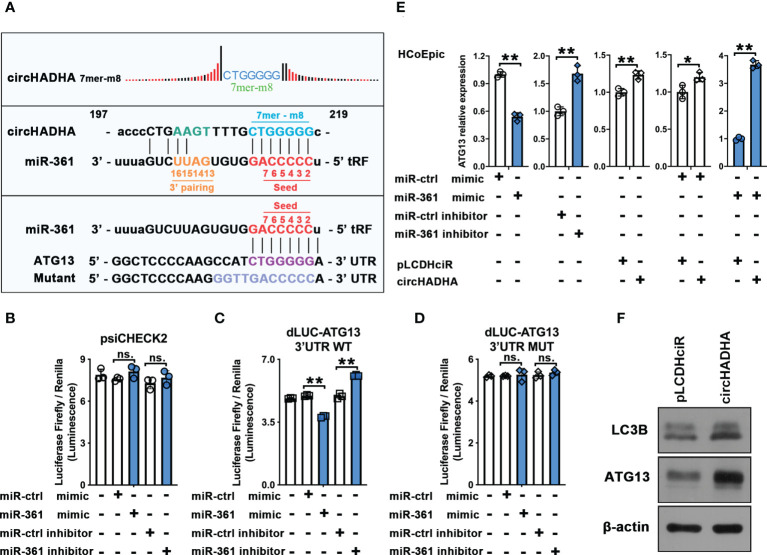
CircHADHA released ATG13 inhibition by competitively recruiting miR-361. **(A)** The sequence of potential binding sites in circHADHA (7mer-m8 position), seed region of miR-361, and 3’UTR of ATG13. **(B–D)** The luciferase reporter between miR-361 and ATG13. **(B)** miR-361 did not regulate luciferase reporter activities of ATG13 in control HCoEpiC. **(C)** miR-361 mimic reduced, whereas miR-361 inhibitor increased, the luciferase activity of ATG13 compared with corresponding controls in HCoEpiC. **(D)** miR-361 did not regulate luciferase reporter activities of ATG13 with mutant in 3’UTR in HCoEpiC. **(E)** The measurement of ATG13 relative expression transfected by miR-361 with or without circHADHA overexpression. (Left panels) miR-361 negatively regulated ATG13 expression compared with corresponding controls in HCoEpiC. (Central panel) circHADHA overexpression increased ATG13 expression compared with corresponding control in HCoEpiC. (Right panels) circHADHA overexpression elevated ATG13 expression regardless of the transfection with miR-361 or control mimic. **(F)** Overexpression of circHADHA increased LC3B II and ATG13 expression at protein level by Western blot. Three independent experiments were performed. **P < 0.01; *P < 0.05; ns, no significant difference.

Potential binding site analysis implied that circHADHA (7mer-m8 position) and ATG13 shared the same complementary seed region of miR-361 at the 5’ end (**Figure 5A**). We inserted complementarity binding sites at 3’UTR of ATG13 into dual-luciferase reporter vectors and measured the luciferase reporter activities with miR-361 cotransfection. **Figure 5B** demonstrated that miR-361 mimic reduced (*P* < 0.01), whereas miR-361 inhibitor increased (*P* < 0.01), the luciferase activities of ATG13 compared with corresponding controls (**Figure 5C**). However, there were no alterations of luciferase reporter activities in the presence of miR-361 mimic or inhibitor cotransfected with the control (**Figure 5B**) or mutant of 3’UTR of ATG13 (**Figure 5D**), respectively. Then, we measured ATG13 expression transfected by miR-361 with or without circHADHA overexpression (**Figure 5E**). miR-361 mimic significantly inhibited the expression of ATG13 in HCoEpiC (*P* < 0.01). While circHADHA overexpression increased ATG13 compared with corresponding control (*P* < 0.01), which was consistent with miR-361 inhibitor transfection in HCoEpiC (*P* < 0.01). However, the expression of ATG13 was elevated in the presence of circHADHA overexpression regardless of the transfection with miR-361 (*P* < 0.01) or control mimic (*P* < 0.05). These results revealed that circHADHA competitively inhibited the combination between miR-361 and 3’UTR of ATG13. Then, we measured the expression of ATG13 and LC3B at the protein level in LPS-injured HCoEpiC. Western blot demonstrated that the expression of ATG13 and LC3B (II/I) was increased in circHADHA-overexpressing HCoEpiC compared to pLCDHciR control cells (**Figure 5F**). As a result, the expression of ATG13 was negatively regulated by the binding of circHADHA to miR-361 competitively in colon epithelial cells.

### CircHADHA regulated autophagy mediated by miR-361 and ATG13

**Figure 6:**
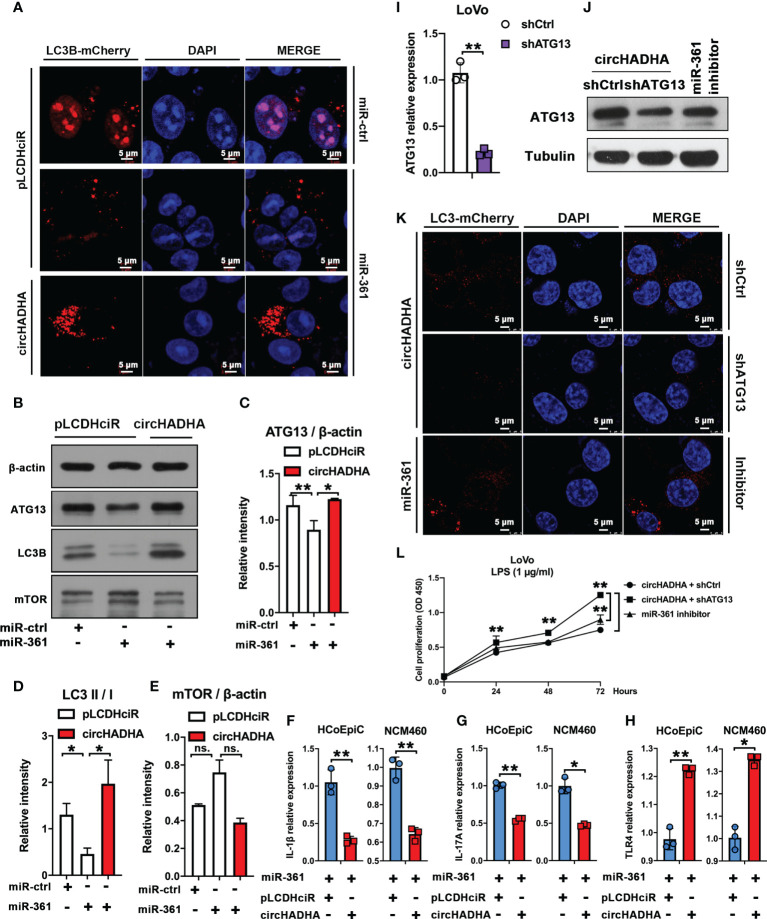
circHADHA regulated autophagy mediated by miR-361 and ATG13. **(A)** The LC3B-mCherry-based autophagy assays in HCoEpiC. In pLCDHciR control cells, miR-361 significantly suppressed autophagy by reducing the production of LC3B-positive autophagosomes with LPS treatment. Overexpression of circHADHA promoted autophagy in the presence of miR-361 treated with LPS in HCoEpiC. mCherry represented LC3B-positive autophagosomes. Cell nuclei were labeled with DAPI. **(B)** The measurement of autophagy-related protein expression by Western blot in LPS-injured HCoEpiC. In the absence of circHADHA, miR-361 inhibited ATG13 and LC3B compared to miR-control. Overexpression of circHADHA increased the expression of ATG13 and LC3B compared to pLCDHciR control transfected with miR-361. The expression of mTOR did not show significant changes with circHADHA/miR-361 treatment. **(C)** The intensity of ATG13 compared to β-actin from Western blot. **(D)** The intensity ratio of LC3B II to I from Western blot. **(E)** The intensity of mTOR compared to β-actin from Western blot. **(F–H)** The inflammatory cytokines were validated in colon epithelial cells with LPS treatment. **(F)** The IL-1β expression was downregulated in circHADHA-overexpressing colon cancer cells compared with control with miR-361 treatment. **(G)** The expression of IL-17A was downregulated in circHADHA-overexpressing colon cancer cells compared with control with miR-361 treatment. **(H)** Overexpression of circHADHA upregulated the expression of TLR4 transfected with miR-361. **(I)** The generation of ATG13 knockdown (shATG13) in LoVo cells. **(J)** The expression of ATG13 was decreased by ATG13 knockdown at the protein level compared to the treatment of shControl or miR-361 inhibitor in circHADHA-overexpressing LoVo cells. **(K)** The LC3B-mCherry-based autophagy assays in LoVo cells. ATG13 knockdown reduced LC3B-positive autophagosomes in circHADHA-overexpressing LoVo cells compared to the transfection of shControl or miR-361 inhibitor with LPS treatment. mCherry represented LC3B-positive autophagosomes. Cell nuclei were labeled with DAPI. **(L)** The measurement of cell viability was performed by CCK-8 assay. Overexpression of circHADHA inhibited proliferation compared with control in LPS-induced LoVo colon cancer cells. ATG13 knockdown increased proliferation in circHADHA-overexpressing LoVo cells compared to the transfection of shControl or miR-361 inhibitor with LPS treatment. Three independent experiments were performed. **P < 0.01; *P < 0.05; ns, no significant difference.

In order to validate the intermediate regulation of miR-361 and ATG13 in circHADHA-induced autophagy, we performed LC3B-based autophagy assays with transfection of miR-361 in HCoEpiC (**Figure 6A**). In pLCDHciR control cells, miR-361 significantly suppressed autophagy by reducing the production of LC3B-positive autophagosomes with LPS treatment. However, circHADHA overexpression promoted LC3B-positive autophagosomes even in the presence of miR-361 treated with LPS in HCoEpiC. Then, we measured ATG13 and LC3B expression at the protein level in LPS-injured HCoEpiC (**Figure 6B**). Western blot demonstrated that miR-361 inhibited ATG13 (**Figure 6C**, *P* < 0.01) and LC3B (**Figure 6D**, *P* < 0.05) compared to miR-control in the absence of circHADHA. However, circHADHA overexpression increased the expression of ATG13 (**Figure 6C**, *P* < 0.05) and LC3B (**Figure 6D**, *P* < 0.05) compared to pLCDHciR control by transfection with miR-361. Additionally, the expression of mTOR did not show significant changes at the protein level with circHADHA/miR-361 treatment (**Figure 6E**). These results showed that miR-361 declined autophagy by inhibiting ATG13, whereas the presence of circHADHA rescued autophagy by competitively recruiting miR-361 from ATG13 in LPS-injured colon epithelial cells. The inflammatory cytokines were validated in colon epithelial cells with LPS treatment. Overexpression of circHADHA decreased the expression of interleukin (IL)-1β (**Figure 6F**, *P* < 0.01, *P* < 0.01) and IL-17A (**Figure 6G**, *P* < 0.01, *P* < 0.05) compared with control in miR-361-treated HCoEpiC and NCM460 cells. Whereas the expression of TLR4 was upregulated in circHADHA-overexpressing HCoEpiC (*P* < 0.01) and NCM460 (*P* < 0.05) cells transfected with miR-361 (**Figure 6H**).

Then, we knocked down ATG13 by shRNA in LoVo (**Figure 6I**, *P* < 0.01). In circHADHA-overexpressing LoVo cells, shATG13 decreased ATG13 expression at the protein level compared with control and miR-361 inhibitor transfection (**Figure 6J**). Autophagy assays demonstrated that shATG13 reduced autophagy in circHADHA-overexpressing LoVo cells compared with control and miR-361 inhibitor transfection with LPS treatment (**Figure 6K**). We also performed CCK-8 assay to evaluate the cell viability in LPS-induced LoVo cells (**Figure 6L**). In the presence of circHADHA, shATG13 increased proliferation compared with shControl (*P* < 0.01). In addition, the inhibitor of miR-361 significantly decreased cell viability compared with knockdown of ATG13 in circHADHA-overexpressing LoVo (*P* < 0.01). Thus, circHADHA promoted autophagy regulated by miR-361 and ATG13 in colon epithelial and cancer cells. Moreover, circHADHA-augmented autophagy impeded cell proliferation mediated by miR-361/ATG13 in colon cancer cells.

### CircHADHA suppressed tumor growth in *xenograft*-bearing nude mice

**Figure 7:**
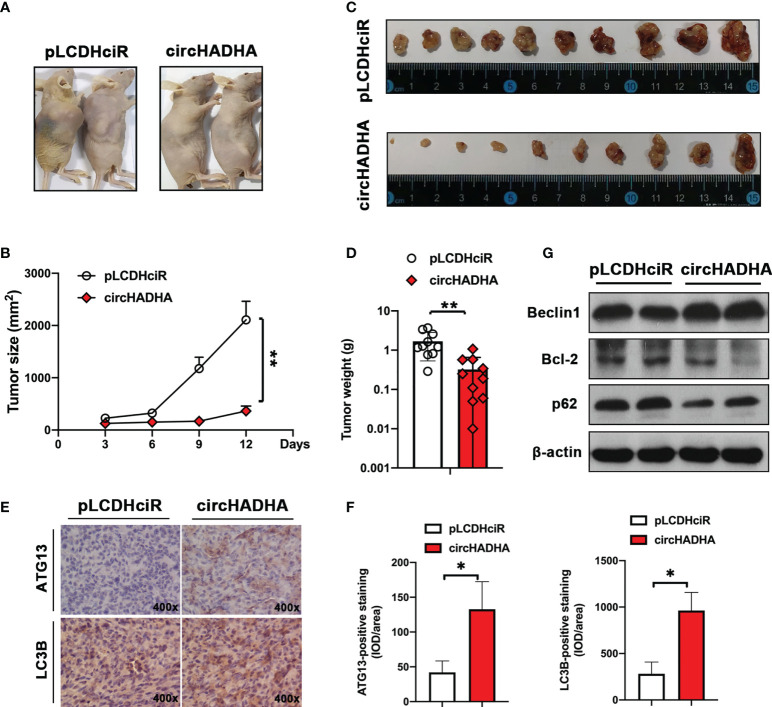
CircHADHA suppressed tumor growth in colon cancer cell-derived xenograft-bearing nude mice. **(A)** The generation of xenograft-bearing nude mice by subcutaneously transplanting with circHADHA-overexpressing or control LoVo cells. **(B)** The tumor size was measured every 3 days. The tumor growth was significantly inhibited in circHADHA-overexpressing LoVo-derived xenograft compared to control tumors. **(C)** Xenograft tumors were isolated from nude mice on the 12th day after xenograft transplantation of circHADHA-overexpressing or control LoVo cells. **(D)** The average weight of xenograft tumors derived from circHADHA-overexpressing or control LoVo cells. Overexpression of circHADHA significantly suppressed xenograft tumor growth. *N* = 10. **(E)** Immunohistochemical staining of ATG13 and LC3B in xenograft tumors derived from circHADHA-overexpressing or control LoVo cells. circHADHA overexpression increased the expression of ATG13 and LC3B in xenograft tumors. **(F)** The staining intensity of ATG13 (left panel) and LC3B (right panel). **(G)** Autophagy-related proteins were measured by Western blot. Beclin1 was expressed higher in xenograft with circHADHA overexpression than in control tumors. Bcl-2 was decreased and p62 was degraded in circHADHA-derived xenograft compared with control tumors. **P < 0.01; *P < 0.05.

We generated xenograft-bearing nude mice by subcutaneously transplanting with circHADHA-overexpressing or control LoVo cells (**Figure 7A**). The tumor size was measured every 3 days. **Figure 7B** showed that circHADHA significantly suppressed xenograft tumor growth (*P* < 0.01). After 12 days, mice were sacrificed, and xenograft tumors were collected (**Figure 7C**). circHADHA significantly reduced the average weight of xenograft tumors (**Figure 7D**, *P* < 0.01). Immunohistochemical staining was performed and demonstrated increasing expression of ATG13 and LC3B in circHADHA-overexpressing xenograft tumors (**Figures 7E, F**). Autophagy-related proteins were measured by Western blot and showed that Beclin1 was expressed more in xenografts with circHADHA overexpression than in control tumors. While Bcl-2 was decreased and p62 was degraded in circHADHA-derived xenografts compared with control tumors (**Figure 7G**).

**Figure 8:**
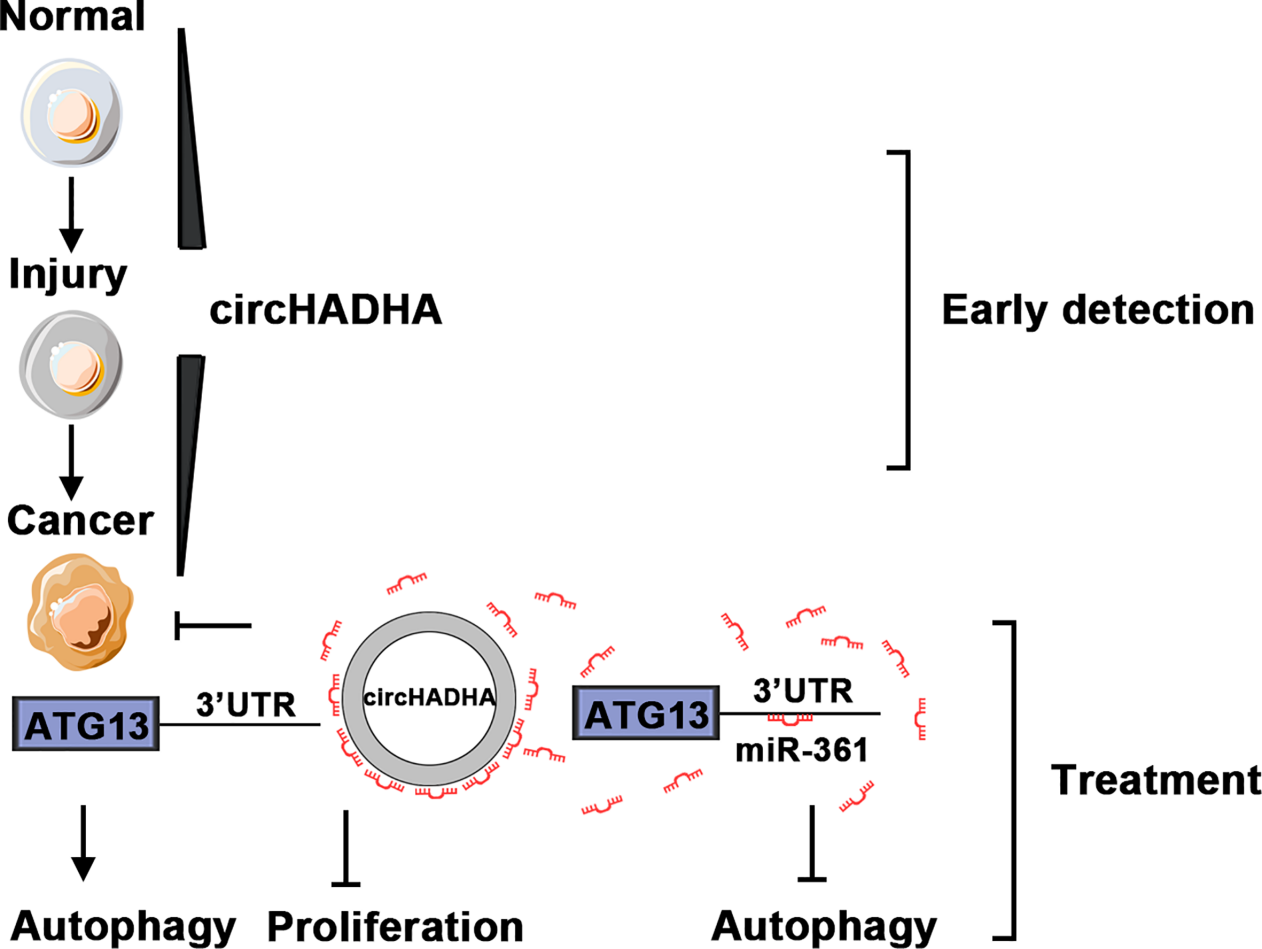
The schematic diagram of circHADHA as a potential biomarker for early detection and treatment of colon cancer. The expression of circHADHA increased in the plasma from colon polyp patients compared to healthy individuals, while it was reduced in the plasma from colon cancer patients compared to polyp patients. The dynamic expressions of plasma circHADHA imply a possibility of a novel noninvasive marker in the early detection of colon cancer. CircHADHA improved autophagy regulated by miR-361 and ATG13 in both colon epithelial and cancer cells that indicated an important role of circHADHA in protecting intestinal epithelial cells from injury. The circHADHA-augmented autophagy impeded cell proliferation mediated by miR-361/ATG13 in colon cancer that depicted a potential of circHADHA to act as a therapeutic target for colon cancer.

Therefore, we provided insights into circHADHA as a potential biomarker and therapeutic target for colon cancer from colon polyps (**Figure 8**). The dynamic expressions of circHADHA in the plasma from healthy individuals and colon polyp and colon cancer patients imply a possibility of a novel noninvasive marker in the early detection of colon cancer. In addition, circHADHA improved autophagy regulated by miR-361 and ATG13 in both colon epithelial and cancer cells, and circHADHA-augmented autophagy impeded cell proliferation in colon cancer cells and colon cancer cell-derived xenograft tumors. These indicates that circHADHA plays an important role in protecting intestinal epithelial cells from injury and may be a target for the treatment of colon cancer.

## Discussion

The conventional oncogenic transformation of colon cancer undergoes a series process from asymptomatic polyp to adenoma-carcinoma. Efficient screening administrations are of benefit to the reduction of the colon cancer-related mortality. Colonoscopy and fecal-based screening, such as FOBT, FIT, and DNA or RNA stool tests, are common clinical applications for the screening and early diagnosis of colon cancer (26). Colonoscopy examination is a standard approach for the detection and therapy of both early cancer and cancer precursor lesions, which is invasive and usually implied after abnormal stool-based screening (27). Fecal screening tests currently lack high sensitivity for precursor lesions of colon cancer, although they are inexpensive and easy to operate (7, 28). Thus, novel noninvasive and economical technologies and biomarkers remain to be explored to combine colonoscopy diagnosis for the prediction of the malignant transformation from premalignancy to colon cancer (8–10). The oncogenic transformation *via* the traditional adenoma-carcinoma pathway is usually driven by mutations in APC, KRAS, SMAD4, and TP53 (4–6). The sessile serrated polyps with high-level microsatellite instability (MSI-H) phenotype are premalignant lesions (29). In order to implement fast and convenient diagnostic strategy to distinguish the potential premalignant risk, more and more tumor markers were identified (30). However, the biomarkers for the prediction of the transformation from normal epithelial-polyps-colon cancer still need to be explored (9).

circRNA is a novel type of noncoding RNA, which is characterized by its circular shape and stable expression (31). circRNA acted as a miRNA sponge, involved in cancer progression. Most circRNAs stem from self-splicing introns of pre-ribosomal RNA (32, 33). In our present study, we found that the significant differentially expressed circRNAs were classified into intronic, exonic, antisense, and intragenic types. Exonic circRNA constitutes the majority. Exons of different genes produce fusion circRNAs that associate with cancerous chromosomal translocations, which are involved in cell transformation, tumor progression, and therapy resistance (34). Indispensably, molecular events are involved in the development of polyp-adenoma-adenocarcinoma progression. The gene mutation and epigenetic regulation are indicated in the whole sequence. circRNA ciRS-7 spatially resolved cellular expression patterns in colon cancer and is highly expressed in stromal cells within the tumor microenvironment (35). The biomarker ciRS-7 reduces epidermal growth factor receptor (EGFR-RAF1) activity in colon cancer patients and promotes growth and metastasis of esophageal squamous cell carcinoma *via* miR-7/HOXB13 (36, 37). In colon cancer tissues, hsa_circ_001988 expression reduced (38) and hsa_circ_001569 negatively correlated with miR-145 as a sponge by attenuating BAG4, E2F5, and FMNL2 expressions (39). In colon cancer cells, hsa_circ_000984 competitively combined with miR-106b as a ceRNA and increased CDK6 expression effectively (40).

circRNAs represent a potential implication in medical practice, which are stably enriched in plasma exosomes and have been reported to be biomarkers for malignant diseases (41–43). circRNAs are enriched in blood much more than corresponding linear RNAs (42). A large-scale identification of metastasis-related circRNAs in colon cancer has been performed to diagnose and investigate the development and metastasis of colon cancer (44). According to published reports, certain circRNAs have been reported as tumor biomarkers in colon cancer by next-generation sequencing. The significant differential expression patterns of circRNAs have been identified between colon cancer and normal cells (44). However, only a few studies reported the candidate circRNAs in precancerous diseases. In the present study, we have analyzed a total of 2,162 human circRNAs and found that the expression pattern of circRNA was altered in colon polyp and colon cancer plasma compared with that in healthy individuals. Potential circRNA candidates were selected as biomarkers to predict malignant progression from colon polyps to cancer.

circRNAs regulate splicing and transcription and act as miRNA sponges or interactors with RNA‐binding proteins (RBPs) (45). circRNAs have been identified as specific targets for the diagnosis and prognosis of colon cancer, involved in the molecular mechanisms of the development and progression of colon cancer (46, 47). Guarnerio et al. (34) reported that well-established cancer-associated chromosomal translocations gave rise to fusion circRNAs, having tumor-promoting properties. circRNAs can also arise from protein-coding genes and act as ceRNAs or miRNA sponges to regulate gene expression (20, 32). In malignant diseases, miRNA sponges have potential effects on oncogenesis and pathway regulation (45). In our present study, we found that the dynamic alterations of circHADHA in colon polyp and colon cancer plasma as well as in healthy individuals have a great potential in predicting colon malignant transformation. We induced LPS injury and performed different assays to confirm the biological function of circHADHA in colon epithelial cells. We found that circHADHA did not have effects on cell viability, colony formation, and apoptosis in colon epithelial cells. However, circHADHA mediated autophagy in colon epithelial cells. We performed an integrative analysis of the ceRNA network between circHADHA, miRNAs, and mRNAs and found a potential interaction between circHADHA, miR-361, and ATG13, which was consistent with autophagy regulation. Luciferase reporter assay, real-time PCR, and competitive inhibition assay demonstrated that circHADHA complementarily bound miR-361 to negatively regulate ATG13 expression, leading to the alteration of autophagy in LPS-injured colon epithelial cells. Furthermore, we performed autophagy assay in colon cancer cells with present or absent circHADHA and showed that circHADHA regulated autophagy *via* the miR-361/ATG13 axis. Therefore, circHADHA acts as a sponge to competitively bind miR-361 and regulate ATG13 expression and autophagy in colon epithelial and cancer cells.

It is interesting that circHADHA overexpression did not affect cell proliferation in normal colon epithelial cells. However, cell viability was significantly inhibited in circHADHA-overexpressing colon cancer cells. And the growth of xenograft tumors was suppressed by circHADHA overexpression. Due to the dynamic alteration of circHADHA expression in plasma from healthy individuals, colon polyp patients, and cancer patients, circHADHA may be a potential candidate for early diagnosis and treatment of colon cancer.

We demonstrated a dynamic alteration of circHADHA in the oncogenic process. circHADHA was upregulated in colon polyp patients compared with healthy individuals, which competitively recruited miR-361 to promote autophagy by releasing ATG13. Whereas it was lowly expressed in colon cancer patients compared with polyp patients, which led to the inhibition of ATG13 by binding miR-361. Although the dynamic expression of circHADHA implies the progress from colon polyps to colon cancers, the findings of this study still have to be seen in light of some limitations. In the present study, we only included patients with sporadic colon polyps and colon cancer, but no patients with familial adenomatous polyposis, Lynch syndrome, and secondary colon cancer. Sporadic colon cancer is a multistep and polygenic disease (48); thus, we cannot rely on a single genetic abnormality or mutation to diagnose colon cancer. The continuous discovery of novel tumor markers, as well as more mechanism studies, will promote the early diagnosis and treatment of colon cancer.

In summary, circHADHA augmented autophagy and suppressed the progression of colon cancer by regulating the autophagy-related gene *via* miR-361. CircHADHA may play important roles in preventing colon polyps from developing into colon cancer.

## Data availability statement

The original contributions presented in the study are included in the article/**Supplementary Material**, further inquiries can be directed to the corresponding author.

## Ethics statement

The studies involving human participants were reviewed and approved by Ethics Committee of First Affiliated Hospital, Jinan University, Guangzhou, China. The patients/participants provided their written informed consent to participate in this study. The animal study was reviewed and approved by Ethics Committee for Animal Research of Jinan University, Guangzhou, China.

## Author contributions

YS and WH designed the experiments. YS, JLi, MT, and YZ performed experiments and analyzed data. YS and JLi performed statistical analyses. YS, JLi, and JLiu collected clinical samples. YS and WH wrote the manuscript. All authors contributed to the article and approved the submitted version.

## Funding

The study was supported by the Medical Science and Technology Foundation of Guangdong Province (A2021306).

## Conflict of interest

The authors declare that the research was conducted in the absence of any commercial or financial relationships that could be construed as a potential conflict of interest.

## Publisher’s note

All claims expressed in this article are solely those of the authors and do not necessarily represent those of their affiliated organizations, or those of the publisher, the editors and the reviewers. Any product that may be evaluated in this article, or claim that may be made by its manufacturer, is not guaranteed or endorsed by the publisher.
