# Supplementary material for: CircHADHA-augmented autophagy suppresses tumor growth of colon cancer by regulating autophagy-related gene via miR-361
Source: Front Oncol. 2022 Oct 13;12:937209. doi: 10.3389/fonc.2022.937209 (PMC9606334; doi:10.3389/fonc.2022.937209)
Supplement: Supplementary file 1 [file Table_1.docx]

**Supplementary Table 1. Differential circRNA candidates**

| CircRNA  ID (hsa) | Polyp vs. normal (> 1.5-fold) | | | Cancer vs. polyp (> 1.5-fold) | | | Type |
| --- | --- | --- | --- | --- | --- | --- | --- |
|  | P-value | Fold | Regulation | P-value | Fold | Regulation |  |
| circ_0015278 | 0.000 | 1.8 | Down | 0.027 | 1.5 | Up | Exonic |
| circ_0029426 | 0.004 | 1.6 | Down | 0.009 | 1.6 | Up | Exonic |
| circ_0029431 | 0.000 | 2.0 | Down | 0.000 | 2.1 | Up | Exonic |
| circ_0066336 | 0.000 | 1.6 | Down | 0.000 | 1.8 | Up | Exonic |
| circ_0008537 | 0.000 | 1.8 | Down | 0.000 | 1.8 | Up | Exonic |
| circ_0001013 | 0.000 | 2.5 | Up | 0.000 | 2.0 | Down | Intragenic |
| circ_0000423 | 0.000 | 1.7 | Up | 0.000 | 2.1 | Down | Exonic |
| circ_0001748 | 0.000 | 1.8 | Up | 0.000 | 1.5 | Down | Intronic |
| circ_0001907 | 0.000 | 1.7 | Up | 0.000 | 1.6 | Down | Intragenic |
| circ_0011462 | 0.000 | 2.0 | Up | 0.000 | 2.2 | Down | Exonic |
| circ_0012265 | 0.000 | 1.6 | Up | 0.000 | 1.7 | Down | Exonic |
| circ_0012850 | 0.000 | 1.7 | Up | 0.000 | 1.8 | Down | Exonic |
| circ_0000091 | 0.000 | 1.7 | Up | 0.000 | 1.6 | Down | Exonic |
| circ_0005758 | 0.000 | 3.3 | Up | 0.000 | 3.7 | Down | Exonic |
| circ_0006520 | 0.000 | 3.7 | Up | 0.000 | 4.2 | Down | Exonic |
| circ_0007681 | 0.000 | 2.4 | Up | 0.000 | 2.4 | Down | Exonic |
| circ_0005783 | 0.000 | 1.5 | Up | 0.000 | 1.7 | Down | Exonic |
| circ_0000669 | 0.000 | 2.1 | Up | 0.000 | 2.0 | Down | Exonic |
| circ_0002631 | 0.000 | 1.6 | Up | 0.000 | 1.6 | Down | Exonic |
| circ_0043949 | 0.000 | 1.8 | Up | 0.000 | 1.8 | Down | Exonic |
| circ_0045932 | 0.000 | 1.6 | Up | 0.000 | 1.9 | Down | Exonic |
| circ_0046188 | 0.000 | 2.8 | Up | 0.000 | 2.6 | Down | Exonic |
| circ_0007117 | 0.000 | 1.7 | Up | 0.000 | 1.8 | Down | Exonic |
| circ_0050102 | 0.000 | 1.8 | Up | 0.000 | 1.7 | Down | Exonic |
| circ_0053063 | 0.000 | 2.5 | Up | 0.000 | 3.3 | Down | Exonic |
| circ_0007422 | 0.000 | 2.5 | Up | 0.000 | 2.4 | Down | Exonic |
| circ_0003915 | 0.000 | 3.4 | Up | 0.000 | 2.9 | Down | Exonic |
| circ_0058495 | 0.000 | 1.7 | Up | 0.000 | 1.9 | Down | Exonic |
| circ_0006704 | 0.000 | 1.7 | Up | 0.000 | 1.6 | Down | Exonic |
| circ_0063756 | 0.000 | 1.6 | Up | 0.000 | 1.6 | Down | Exonic |
| circ_0008199 | 0.000 | 2.2 | Up | 0.000 | 1.8 | Down | Exonic |
| circ_0005870 | 0.000 | 1.6 | Up | 0.000 | 1.7 | Down | Exonic |
| circ_0066887 | 0.000 | 1.8 | Up | 0.000 | 1.7 | Down | Exonic |
| circ_0007883 | 0.000 | 2.8 | Up | 0.000 | 3.2 | Down | Exonic |
| circ_0075829 | 0.000 | 1.5 | Up | 0.000 | 1.5 | Down | Exonic |
| circ_0084678 | 0.000 | 1.7 | Up | 0.000 | 1.5 | Down | Exonic |
| circ_0089169 | 0.000 | 1.7 | Up | 0.000 | 2.0 | Down | Exonic |
| circ_0092312 | 0.000 | 1.6 | Up | 0.000 | 1.7 | Down | Intronic |
