# Supplementary material for: CircHADHA-augmented autophagy suppresses tumor growth of colon cancer by regulating autophagy-related gene via miR-361
Source: Front Oncol. 2022 Oct 13;12:937209. doi: 10.3389/fonc.2022.937209 (PMC9606334; doi:10.3389/fonc.2022.937209)
Supplement: Supplementary file 2 [file Table_2.docx]

**Supplementary Table 2. Predicted miRNA binding sites in circHADHA**

| MiRNA candidates | Accession | Sequence | Predicted binding sites in circHADHA | |
| --- | --- | --- | --- | --- |
|  |  |  | Range | Seed match |
| hsa-miR-26a-1 | MIMAT0004499 | ccuauucuugguuacuugcacg | (42, 66) | 7mer-m8 |
|  |  |  | (145, 168) | 7mer-m8 |
| hsa-miR-26a-2 | MIMAT0004681 | ccuauucuugauuacuuguuuc | (42, 66) | 7mer-m8 |
|  |  |  | (146, 168) | 7mer-m8 |
| hsa-miR-361 | MIMAT0004682 | ucccccaggugugauucugauuu | (197, 219) | 7mer-m8 |
| hsa-miR-214 | MIMAT0000271 | acagcaggcacagacaggcagu | (254, 276) | 7mer-m8 |
